# Complete Genome Sequence of *Pluralibacter gergoviae* FB2, an *N*-Acyl Homoserine Lactone-Degrading Strain Isolated from Packed Fish Paste

**DOI:** 10.1128/genomeA.01276-14

**Published:** 2014-12-11

**Authors:** Kok-Gan Chan, Kok Keng Tee, Wai-Fong Yin, Jia-Yi Tan

**Affiliations:** a Division of Genetics and Molecular Biology, Institute of Biological Sciences, Faculty of Science, University of Malaya, Kuala Lumpur, Malaysia; b Centre of Excellence for Research in AIDS (CERiA), Department of Medicine, Faculty of Medicine, University of Malaya, Kuala Lumpur, Malaysia

## Abstract

*Pluralibacter gergoviae* FB2, a bacterial strain isolated from packed food, has been found to exhibit quorum-quenching properties. Hence, we report the first, complete genome of *P. gergoviae* sequenced using the Pacific Biosciences single-molecule, real-time (SMRT) platform.

## GENOME ANNOUNCEMENT

Microbial food spoilage has been reported to cause alarming economic losses in the food industry (1). In recent years, various studies have associated the event to quorum sensing (QS), a cell-to-cell communication strategy adapted by a wide range of proteobacteria (2). For instance, QS by means of *N-*acyl-homoserine lactone (AHLs) has been linked to the formation of biofilms, a known form of chronic contamination on food processing surfaces (3), as well as a number of proteolytic and lipolytic phenotypes (4, 5).

*Pluralibacter gergoviae*, formerly named as *Enterobacter gergoviae*, are Gram-negative, facultative anaerobic straight rods of 0.6–1.0 µm × 1.5–2.5 µm in size (6). As the genus name implies, this organism has been isolated from a wide range of sources (7). In this study, *P. gergoviae* FB2 was isolated from refrigerated packed fish paste, which is popular in East and Southeast Asia. A routine quorum-quenching (QQ) screening assay was performed as previously described (8), which indicated that this strain is able to degrade AHLs.

Genomic DNA of *P. gergoviae* FB2 was extracted using the MasterPure DNA purification kit (Epicentre, Inc., Madison, WI, USA) prior to sequencing via the Pacific Biosciences single-molecule, real-time (SMRT) sequencer (Pacific Biosciences, Menlo Park, CA, USA) sequencer. Routine quality checking on the DNA sample was performed using the NanoDrop spectrophotometer (Thermo Scientiﬁc, Waltham, MA, USA), the Qubit version 2.0 ﬂuorometer (Life Technologies, Carlsbad, CA, USA), and gel electrophoresis. A SMRTbell library was prepared from sheared genomic DNA according to the 20-kb template library preparation workflow using P5 chemistry. The prepared library was sequenced on two SMRT cells, yielding output data with an average genome coverage of 93.65×. A single circular contig was obtained from *de novo* assembly of the insert reads performed with the Hierarchical Genome Assembly Process (HGAP) algorithm in the SMRT Portal (version 2.1.1). According to annotation via the NCBI Prokaryotic Genome Annotation Pipeline (PGAAP) (9), the genome size is 5.49 Mb with 59.1% GC content consisting of 4,856 open reading frames (ORFs), 4,692 protein-coding sequences, 22 rRNAs, 84 tRNAs, 1 other rRNA, and 57 pseudogenes.

Analysis of the genomic data via RAST (Rapid Annotation using Subsystem Technology) (10) revealed the presence of a putative AHL hydrolase gene. The sequence is 792 bp in length, encoding 264 amino acids that show 87% similarity to a reported *attM* gene of *Agrobacterium tumefaciens* (11). We hope that the finding of an AHL-degrading gene in *P. gergoviae* FB2 will provide an insight into the role of QQ in interspecies competition in the context of food spoilage.

### Nucleotide sequence accession number.

This complete genome project has been deposited in DDBJ/ENA/GenBank under the accession number CP009450. The version described in this paper is the first version.

## References

[1] Kumar CG Anand SK 1998 Significance of microbial biofilms in food industry: a review. Int. J. Food Microbiol. 42:9–27. 10.1016/S0168-1605(98)00060-9. 9706794

[2] Blana VA Nychas GJ 2014 Presence of quorum sensing signal molecules in minced beef stored under various temperature and packaging conditions. Int. J. Food Microbiol. 173:1–8. 10.1016/j.ijfoodmicro.2013.11.028. 24380750

[3] Bai AJ Rai VR 2011 Bacterial quorum sensing and food industry. Comp. Rev. Food Sci. Food Saf. 10:184–194. 10.1111/j.1541-4337.2011.00150.x.

[4] Jay JM Vilai JP Hughes ME 2002 Profile and activity of the bacterial biota of ground beef held from freshness to spoilage at 5–7°C. Food Microbiol. 81:105–111. 10.1016/S0168-1605(02)00189-7. 12457584

[5] Christensen AB Riedel K Eberl L Flodgaard LR Molin S Gram L Givskov M 2003 Quorum-sensing-directed protein expression in *Serratia proteamaculans* B5a. Microbiology 149:471–483. 10.1099/mic.0.25575-0. 12624209

[6] Brady C Cleenwerck I Venter S Coutinho T De Vos P 2013 Taxonomic evaluation of the genus *Enterobacter* based on multilocus sequence analysis (MLSA): proposal to reclassify *E. nimipressuralis* and *E. amnigenus* into *Lelliottia* gen. nov. as *Lelliottia nimipressuralis* comb. nov. and *Lelliottia amnigena* comb. nov., respectively, *E. gergoviae* and *E. pyrinus* into *Pluralibacter* gen. nov. as *Pluralibacter gergoviae* comb. nov. and *Pluralibacter pyrinus* comb. nov., respectively, *E. cowanii*, *E. radicincitans*, *E. oryzae* and *E. arachidis* into *Kosakonia* gen. nov. as *Kosakonia cowanii* comb. nov., *Kosakonia radicincitans* comb. nov., *Kosakonia oryzae* comb. nov. and *Kosakonia arachidis* comb. nov., respectively, and *E. turicensis*, *E. helveticus* and *E. pulveris* into *Cronobacter* as *Cronobacter zurichensis* nom. nov., *Cronobacter helveticus* comb. nov. and *Cronobacter pulveris* comb. nov., respectively, and emended description of the genera *Enterobacter* and *Cronobacter*. Syst. Appl. Microbiol. 36:309–319. 10.1016/j.syapm.2013.03.005. 23632228

[7] Périamé M Pagès JM Davin-Regli A 2014 *Enterobacter gergoviae* adaptation to preservatives commonly used in cosmetic industry. Int. J. Cosmet. Sci. 36:386–395. 10.1111/ics.12140. 24828151

[8] Chan KG Atkinson S Mathee K Sam CK Chhabra SR Cámara M Koh CL Williams P 2011 Characterization of *N*-acylhomoserine lactone-degrading bacteria associated with the *Zingiber officinale* (ginger) rhizosphere: co-existence of quorum quenching and quorum sensing in *Acinetobacter* and *Burkholderia*. BMC Microbiol. 11:51. 10.1186/1471-2180-11-51. 21385437 PMC3062576

[9] Pruitt KD Tatusova T Brown GR Maglott DR 2012 NCBI Reference sequences (RefSeq): current status, new features and genome annotation policy. Nucleic Acids Res. 40:D130–D135. 10.1093/nar/gkr1079. 22121212 PMC3245008

[10] Aziz RK Devoid S Disz T Edwards RA Henry CS Olsen GJ Olson R Overbeek R Parrello B Pusch GD Stevens RL Vonstein V Xia F 2012 SEED servers: high-performance access to the SEED genomes, annotations, and metabolic models. PLoS One 7:e48053. 10.1371/journal.pone.0048053. 23110173 PMC3480482

[11] Haudecoeur E Tannières M Cirou A Raffoux A Dessaux Y Faure D 2009 Different regulation and roles of lactonases AiiB and AttM in *Agrobacterium tumefaciens* C58. Mol. Plant Microbe Interact. 22:529–537. 10.1094/MPMI-22-5-0529. 19348571

